# Changes in serum fibronectin levels predict tumor recurrence in patients with early hepatocellular carcinoma after curative treatment

**DOI:** 10.1038/s41598-020-78440-w

**Published:** 2020-12-04

**Authors:** Sun Ah Kim, Eun Ju Cho, Sungyoung Lee, Young Youn Cho, Boram Kim, Jung-Hwan Yoon, Taesung Park

**Affiliations:** 1 The Research Institute of Basic Sciences, Seoul National University, Seoul, Korea; 2 Department of Internal Medicine and Liver Research Institute, Seoul National University College of Medicine, 101 Daehak-ro, Jongno-gu, Seoul, 03080 Republic of Korea; 3 Center for Precision Medicine, Seoul National University Hospital, Seoul, Korea; 4 Department of Internal Medicine, Chung-Ang University Hospital, Seoul, Korea; 5 Interdisciplinary Program in Bioinformatics, Seoul National University, Seoul, Korea; 6 Department of Statistics, Seoul National University, 1 Gwanak-ro, Gwanak-gu, Seoul, 08826 Republic of Korea

**Keywords:** Liver cancer, Tumour biomarkers

## Abstract

Fibronectin, a matrix glycoprotein aberrantly expressed in various tumor cells, is a known candidate biomarker for the early diagnosis of hepatocellular carcinoma (HCC). In this study, we investigated whether serum fibronectin levels could predict tumor recurrence in patients with early-stage HCC after curative treatment. A total of 83 patients who showed complete response after initial curative treatment were included. The levels of serum fibronectin at baseline and 4–6 weeks after initial treatment were analyzed with regard to their associations with recurrence. Multivariate logistic regression analyses were performed to construct a prognostic nomogram. Baseline fibronectin levels were not significantly correlated with tumor size, number, stage, and serum α-fetoprotein levels. However, decrease in serum fibronectin levels after treatment was significantly associated with reduced HCC recurrence in multivariate logistic regression (odds ratio, 0.009; *p* < 0.001). Furthermore, a nomogram consisting of gender and changes in serum fibronectin showed a good discriminatory capability for the prediction of HCC recurrence with an area under the receiver-operating curve of 0.87. In conclusion, changes in serum fibronectin levels may be a surrogate indicator for assessment of treatment response in patients with early HCC after curative treatment.

## Introduction

Hepatocellular carcinoma (HCC) is the fifth most common cancer worldwide and the main etiology of mortality in patients with chronic liver disease^1^. With the implementation of surveillance in high-risk patients and improvement of treatment modalities, the chances of curative treatment have increased^2,3^. However, the high frequency of tumor recurrence in the remnant liver even after curative treatment still limits long-term prognosis of patients. Therefore, for selecting appropriate therapies and predicting prognosis, it is important to develop biomarkers that can predict tumor recurrence after curative treatment.

Fibronectin is a matrix glycoprotein which is aberrantly expressed in various tumors, and promotes tumor progression, metastasis and treatment resistance via activation of a number of downstream signaling pathways^4,5^. Previously, we reported the potential of serum fibronectin as a diagnostic biomarker for HCC^6^. The diagnostic performance of serum fibronectin exceeded that of the current marker, α-fetoprotein (AFP), for the detection of early-stage HCC from liver cirrhosis. Furthermore, serum fibronectin levels decreased after treatment in patients with early-stage HCC who showed 6-month disease-free status after curative treatment, suggesting that levels of fibronectin might serve as a potential predictive (prediction of short-term therapeutic response) biomarker for HCC. However, fibronectin has been little tested as a prognostic marker in HCC patients. The present study aimed to evaluate whether changes in serum fibronectin levels could predict prognosis of patients with early-stage HCC according to different risks of disease recurrence after curative-intent treatment.

## Materials and methods

### Study population

Between December 2012 and April 2017, a total of 293 patients with newly diagnosed HCC who had evaluable paired pre- and post-treatment serum samples were identified from a prospective HCC cohort registry of the Seoul National University Hospital (Seoul, Republic of Korea). Among them, 83 patients with very early- or early-stage HCC according to the Barcelona Clinic for Liver Cancer (BCLC) staging system, who showed complete response after initial treatment (i.e. surgical resection, ablation therapy, or transarterial chemoembolization (TACE)), were included in this study. Patients who underwent liver transplantation were excluded^1^.

The diagnosis of HCC was based on clinicoradiological criteria or histological examination, with regard to the practice guidelines from the European Association for the Study of the Liver or the American Association for the Study of Liver Diseases^1,7^. Treatment modality was discussed and decided at a multidisciplinary meeting. In general, surgical resection was performed in patients with Child–Pugh class A status with less than a 25% indocyanine green retention rate at 15 min and an anatomically resectable tumor. Patients who were unable or unwilling to undergo major surgery received ablation therapy. The TACE was performed in patients who were unsuitable for resection or ablation because of liver function, comorbidity or technical infeasibility. Treatment response was assessed by three-phase dynamic computed tomogram at one month after initial treatment, and then every 2–3 months until recurrence. Complete response after initial treatment and HCC recurrence were defined according to the modified Response Evaluation Criteria in Solid Tumors criteria^8^.

The protocol of the present study conformed to the ethical guidelines of the World Medical Association Declaration of Helsinki and was approved by the Institutional Review Board of Seoul National University Hospital (IRB No. 0506-150-005). All study participants provided written informed consent.

### Measurement of serum fibronectin

Overnight-fasting serum samples were obtained from all patients before and after 4–6 weeks of initial treatment, and stored at − 80 °C. Serum fibronectin was measured using standard commercial kits (Origene, Rockville, MD, USA) by a researcher who was blind to the study endpoint. Each sample was assayed in triplicate, and the mean value used in the analysis.

### Statistical analysis

Continuous variables are presented as medians and ranges. The Mann–Whitney U test was used to analyze differences between the different groups. The *χ*^2^ test or Fisher’s exact test were used for categorical data.

To develop a prognostic nomogram to predict likelihood of HCC recurrence after complete response, we applied simple and multiple logistic regression analyses. Patients who developed HCC recurrence were classified as the recurrence group, and the others were classified as the non-recurrence group. Each of the variables was examined for its association with outcome. The level of fibronectin was log-transformed to alleviate skewness, and denoted by log(fibronectin). The differences between pre- and post-treatment AFP (denoted by AFP_Diff_) and log(fibronectin) (denoted by log(fibronectin)_Diff_) were also considered as predictor variables. Statistical significance was considered to be *p* < 0.05.

Prognostic nomogram models were constructed using multivariate logistic regression analyses for all possible combinations of predictor variables. To select the best nomogram model, we considered two types of quality measures: Akaike information criterion (AIC) and an area under the receiver-operating curve (AUC)^9^. Because the total number of samples was small, we first selected the candidate models with the lowest AIC for each number of variables used in a model. Then for the models selected based on AIC, we estimated the prediction ability of the models by calculating AUC.

We employed a leave-pair-out cross-validation (LPOCV) approach for calculating AUC in order to decrease the risk of overfitting due to the small sample size^10^. Analogous to LOOCV (leave-one-out cross-validation), LPOCV uses each possible case–control pair of the training set as the validation data. The LPOCV approach is known to provide an almost unbiased and more reliable estimate of expected AUC performance in a small-sample setting^11^. In this study, the AUC performance for each candidate model was calculated using the method of Airola et al.^11^. If ${X}_{+}$ and ${X}_{-}$ be the sets of recurrence and non-recurrence groups, respectively; then the AUC performance with LPOCV was calculated as follows:

$$\frac{1}{\left|{X}_{+}\right|\left|{X}_{-}\right|}\sum_{{x}_{i}\in {X}_{+}}\sum_{{x}_{j}\in {X}_{-}}H\left({f}_{\left\{i,j\right\}}\left({x}_{i}\right)-{f}_{\left\{i,j\right\}}\left({x}_{j}\right)\right),$$

where ${f}_{\left\{i,j\right\}}$ denotes a classifier trained without ${x}_{i}$ and ${x}_{j}$, and $H$ is the Heaviside step function defined as $H\left(x\right)=1$ if $x>0$, $H\left(x\right)=0$ if $x<0$, and $H\left(x\right)=1/2$ if $x=0$. All statistical analyses were conducted with R software (Version 3.4.4, http://www.R-project.org).

## Results

### Baseline characteristics

The demographic and clinical characteristics of the patients are shown in Table 1. During a median follow-up period of 40.6 months (range 7.3–70.6 months), 39 (46.9%) patients developed HCC recurrence after curative-intent treatment. The median time to recurrence was 39.8 months (95% confidence interval, 25.3 months–not estimable). Among them, 34 (87.2%) patients developed intrahepatic distant recurrence after treatment, including resection (n = 13), RFA (n = 14), and TACE (n = 18). Local recurrence developed in five (12.8%) patients, including two treated by RFA and three underwent TACE (Supplementary Table 1).

**Table 1:** Clinicopathologic characteristics of the patients.

Variables	Total (n = 83)	Non-recurrence (n = 44)	Recurrence (n = 39)	*p*
**Age, median (range)**	62 (38–82)	62 (40–82)	59 (38–77)	0.43
**Gender, no. (%)**				< 0.001
Female	17 (20.5)	16 (36.4)	1 (2.6)	
Male	66 (79.5)	28 (63.6)	38 (97.4)	
**Etiology, no. (%)**				0.25
HBV	64 (77.1)	36 (81.8)	28 (71.8)	
HCV	9 (10.8)	2 (4.5)	7 (17.9)	
Alcohol	6 (7.2)	4 (9.1)	2 (5.1)	
Others	4 (4.8)	2 (4.5)	2 (5.1)	
**Initial treatment, no. (%)**				0.66
Resection	30 (36.1)	17 (38.6)	13 (33.3)	
Ablation therapy	35 (42.2)	19 (43.2)	16 (41.0)	
TACE	18 (21.7)	8 (18.2)	10 (25.6)	
**FIB-4, no. (%)**				0.82
≤ 3.25	49 (59.0)	25 (56.8)	24 (61.5)	
> 3.25	34 (41.0)	19 (43.2)	16 (38.5)	
**Platelet, × 10**^**3**^**/mL, median (range)**	145 (32–330)	137 (38–330)	146 (32–249)	0.52
**ALT, IU/L, median (range)**	38 (9–238)	37 (9–238)	38 (14–165)	0.39
**Tumor number, median (range)**	1 (1–3)	1 (1–3)	1 (1–2)	0.99
**Maximal tumor size, median (range)**	2.4 (0.5–9.0)	2.3 (0.5–9.0)	2.6 (1.0–5.8)	0.38
**BCLC stage**				1.00
0	28 (33.7)	15 (34.1)	13 (33.3))	
A	55 (66.3)	29 (65.9)	26 (66.7)	
Microvascular invasion*	11 (36.7)	7 (41.2)	4 (30.8)	0.71
Encapsulation*	23 (76.7)	14 (82.4)	9 (69.2)	0.67
Major Edmondson–Steiner grade*				0.45
I, II	19 (63.3)	12 (70.6)	7 (53.8)	
III, IV	11 (36.7)	5 (29.4)	6 (46.2)	
Worst Edmondson–Steiner grade*				0.71
I, II	10 (33.3)	5 (29.4)	5 (38.5)	
III, IV	20 (66.7)	12 (70.6)	8 (61.5)	
**Serum AFP, ng/mL, median (range)**				
Pre-treatment	7.1 (1.0–76,200.0)	5.2 (1.0–76,200.0)	14.4 (1.8–4210.0)	0.25
Post-treatment	4.6 (0.5–127.2)	4.5 (0.5–32.5)	5.7 (1.9–127.2)	0.18
Difference	1.5 (− 24.5 to 76,190.0)	1.2 (− 17.2 to 76,190.0)	2.6 (− 24.5 to 4204.1)	0.33
**Serum fibronectin, ng/mL, median (range)**				
Pre-treatment	4.4 (1.4–22.6)	6.2 (1.7–19.7)	3.9 (1.4–22.6)	0.02
Post-treatment	4.7 (1.3–22.9)	4.4 (1.3–22.9)	5.8 (1.5–16.4)	0.03
Difference	0.3 (− 10.6 to 12.2)	1.1 (− 4.8 to 12.2)	− 0.9 (− 10.6 to 8.2)	< 0.001

*AFP* α-fetoprotein, *ALT* alanine aminotransferase, *BCLC* Barcelona Clinic Liver Cancer, *HBV* hepatitis B virus, *HCV* hepatitis C virus, *TACE* transarterial chemoembolization.

*Assessed in patients who underwent resection.

Patients in the recurrence group had similar characteristics compared with the non-recurrence group, except that males were predominant in the recurrence group (*p* < 0.001). The levels of pre-treatment, post-treatment and difference of serum AFP did not significantly differ between the two groups. However, the recurrence group had significantly higher fibronectin levels after treatment, even though their pre-treatment levels were lower than those of the non-recurrence group. Elevation of fibronectin levels after therapy occurred in the recurrence group whereas the opposite was observed in the non-recurrence group. The pattern of changes in serum AFP and fibronectin levels did not significantly differ according to the recurrence pattern (Supplementary Table 1).

### Levels of serum fibronectin according to clinical characteristics

The associations between clinical factors and the levels of serum fibronectin were analyzed. The levels of fibronectin were not significantly different according to age, gender, etiologies of underlying liver disease and alanine aminotransferase (ALT) levels, but showed a negative correlation with platelet levels with marginal significance (r =  − 0.19, *p* = 0.09). With respect to tumor factors, fibronectin levels were not significantly correlated with maximum tumor size, tumor number, BCLC stage, microvascular invasion, encapsulation, histologic grade, and AFP levels.

### Development of nomogram for predicting HCC recurrence

In the univariate logistic regression analysis, gender and log(fibronectin)_Diff_ showed a significant association with HCC recurrence with odds ratios of 21.71 (*p* = 0.004) and 0.009 (*p* < 0.001), respectively (Table 2). The univariate AUC values for gender and log(fibronectin)_Diff_ were 0.669 and 0.816, respectively. The other predictors were not significant in univariate analysis.

**Table 2:** Univariate logistic regression analyses for recurrence of hepatocellular carcinoma.

Variables	Odds ratio	*p*
**Age**	0.98	0.33
**Gender**		
Female	–	–
Male	21.71	0.004
**Treatment**		
Resection	–	–
Ablation therapy	0.91	0.85
TACE	1.48	0.50
**Etiology**		
HBV	–	–
HCV	4.54	0.10
Alcohol	0.91	0.94
Others	1.82	0.58
**FIB-4**		
≤ 3.25	–	–
> 3.25	0.82	0.66
**Platelet, × 10**^**3**^**/μL**	1.00	0.30
**ALT, IU/L**	1.00	0.62
**Tumor number**	0.91	0.85
**Maximal tumor size**	1.11	0.52
**BCLC stage**		
0	–	–
A	1.03	0.94
Microvascular invasion*	0.64	0.56
Encapsulation*	0.48	0.41
Major Edmondson–Steiner grade*		
I, II	–	–
III, IV	2.06	0.35
Worst Edmondson–Steiner grade*		
I, II	–	–
III, IV	0.67	0.60
**AFP, ng/mL**		
Pre-treatment	1.00	0.67
Post-treatment	1.04	0.19
Difference	1.00	0.68
**Fibronectin (log value)**		
Pre-treatment	0.60	0.10
Post-treatment	1.14	0.67
Difference^†^	0.009	< 0.001

*AFP* α-fetoprotein, *ALT* alanine aminotransferase, *BCLC* Barcelona Clinic Liver Cancer, *TACE* transarterial chemoembolization.

*Assessed in patients who underwent resection.

^†^Defined by subtracting log-transformed post-treatment fibronectin value from log-transformed pre-treatment value.

**Figure 1:**
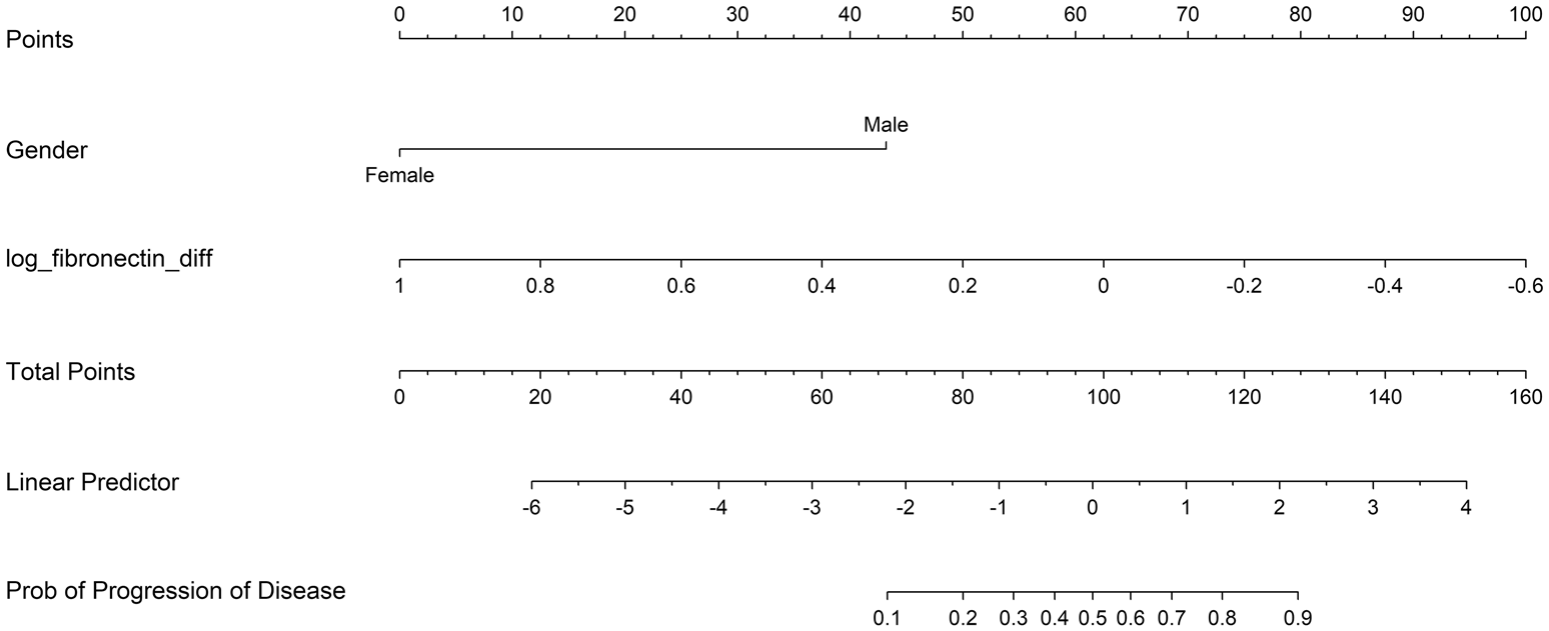
Nomogram to predict likelihood of hepatocellular carcinoma recurrence after curative treatment.

We further developed a model for predicting HCC recurrence after curative treatment. The multivariate logistic regression analysis results of the final model consisted of two variables: gender and log(fibronectin)_Diff_ (Table 3). The AIC and the AUC of the model were 78.44 and 0.87, respectively. Figure 1 shows a nomogram-based scoring system for determining likelihoods of HCC recurrence after curative treatment, which integrated the aforementioned variables. The automated calculation of the score is available at http://statgen.snu.ac.kr/software/nomogram-hcc/.

**Table 3:** Multivariate logistic regression analysis results for the selected variables in the final nomogram.

Variables	Odds ratio	*p*	AIC	AUC
Male	28.77	5.2 $\times$ 10^–3^	78.44	0.87
Log(fibronectin)_Diff_	5.8 $\times$ 10^–3^	1.0 $\times$ 10^–4^		

*AIC* Akaike’s information criteria, *AUC* area under the receiver-operating curve.

Considering the potential effects of incomplete treatment on the levels of fibronectin, we performed additional analysis after excluding subjects with local tumor progression. Sensitivity analysis also revealed similar results (Supplementary Tables 2 and 3).

## Discussion

In this study, we developed a nomogram tailored to the individual patient which reliably generated numerical probabilities of recurrence after curative treatment. This tool is simple and easy-to-use, integrating three predictive variables including changes in serum biomarker levels and gender. Considering the lack of consensus on the follow-up strategies for detection of recurrent HCC after curative treatment^7^, this nomogram enabled patients to be easily monitored on an individual basis.

Fibronectin, which exists in circulating and cellular forms, is upregulated in several types of cancers and its expression positively correlates with invasion and metastasis of cancers^12,13^. Deposition of fibronectin into the extracellular matrix has been shown to support tumor cell proliferation and angiogenesis, which are crucial steps in completion of metastasis^14^. A previous study reported that fibronectin accumulated around HCC nodules and the surrounding fibrous capsule^15^, which might be relevant to the malignant phenotype of tumor cells in favoring cell proliferation and migration^16,17^. Furthermore, a recent study showed that fibronectin enhances migration of HCC circulating tumor cells via regulation of integrin B1 and SLUG^18^. In addition, HCC cells produce and shed surface fibronectin, contributing to the increasing levels of fibronectin in plasma or ascitic fluid^19^. Collectively, these findings suggest that fibronectin is strongly related to HCC.

In this study, we sought to evaluate the clinical value of serum fibronectin levels in HCC. Whereas pre- and post-treatment serum fibronectin levels were not useful for predicting tumor recurrence after curative treatment, the change in serum fibronectin levels was a prognostic factor related to outcome, which was similarly observed in the association between changes in AFP levels and outcome. It is well known that only a small proportion of HCCs (10–20%) at an early stage present with increased AFP levels at baseline, leading to suboptimal prognostic value in early HCC^1^. Because only patients with very early or early HCC were included in this study, the low levels of fibronectin might contribute to low statistical power to detect associations with outcomes. However, changes in serum fibronectin levels were significantly associated with tumor recurrence after curative treatment, suggesting that serial measurements of fibronectin over time may aid clinical judgment.

Serum AFP is frequently measured in routine clinical practice, not only for diagnosis or pre-treatment prognostication but also for prediction of treatment response, because it is thought to constantly reflect tumor burden and biology^20,21^. Previous studies suggested that tumor response may be detected earlier by changes in AFP than by imaging studies, and changes in AFP are strong prognostic factors for survival outcomes with better consistency than radiologic response-based criteria^22,23^. Changes in serum AFP have also been reported as a valuable predictive factor of disease progression and survival in patients with HCC who underwent resection, locoregional therapies or systemic chemotherapy^20,22–24^. Therefore, response evaluation through serial measurement of AFP may be a reliable surrogate marker to predict prognosis after curative treatment.

Interestingly, male sex was associated with tumor recurrence and poor survival after curative treatment in our model. Previous studies reported that the interaction between androgen axis and HBx increased the risk of HCC in male chronic hepatitis B patients^25–27^. The male predominance of HCC recurrence after curative treatment in our study may be at least partially linked with this hepatocarcinogenic feature. Further studies are required to confirm the sex disparity in the prognosis after curative treatment for HCC.

The present study has some limitations. First, the study was performed at a single institution in patients with very early- or early-stage HCC. Therefore, our study may lack a representative sampling of patients with intermediate-to-advanced HCC. Additional multicenter prospective studies on patients with various stages of HCC are warranted for generalizability of the results. Second, some patients had a relatively short follow-up period, which might lead to an underreported recurrence rate. In addition, the association of fibronectin with overall survival was not fully investigated because of the small number of events. Further follow-up of our cohort will clarify this association. Third, tumors might not be completely controlled after locoregional therapies in the local recurrence group which could affect post-treatment fibronectin levels, although sensitivity analysis excluding the local recurrence group showed similar results. Further studies performed in larger numbers of patients treated by resection are needed to validate the prognostic value of fibronectin.

In conclusion, changes in serum fibronectin levels may be a surrogate indicator for assessment of treatment response in HCC. Our biomarker-based nomogram might be helpful for predicting tumor recurrence in patients with early HCC undergoing curative treatment.

## Supplementary information

Supplementary information.

## Abbreviations

AFP: α-Fetoprotein
AIC: Akaike’s information criterion
ALT: Alanine aminotransferase
AUC: Area under the receiver-operating curve
BCLC: Barcelona Clinic Liver Cancer
HCC: Hepatocellular carcinoma
LPOCV: Leave-pair-out cross-validation
TACE: Transarterial chemoembolization
