# Supplementary material for: Changes in serum fibronectin levels predict tumor recurrence in patients with early hepatocellular carcinoma after curative treatment
Source: Sci Rep. 2020 Dec 4;10:21313. doi: 10.1038/s41598-020-78440-w (PMC7719187; doi:10.1038/s41598-020-78440-w)
Supplement: Supplementary file 1 — Supplementary information. [file 41598_2020_78440_MOESM1_ESM.docx]

**Changes in serum fibronectin levels predict tumor recurrence in patients with early hepatocellular carcinoma after curative treatment**

Sun Ah Kim^1*^, Eun Ju Cho^2*^, Sungyoung Lee^3^, Young Youn Cho^4^, Boram Kim^5^, Jung-Hwan Yoon^2^**, and Taesung Park^5, 6^**

*^1^The Research Institute of Basic Sciences, Seoul National University, Seoul, Korea*

*^2^Department of Internal Medicine and Liver Research Institute, Seoul National University College of Medicine, Seoul, Korea*

*^3^Center for Precision Medicine, Seoul National University Hospital, Seoul, Korea*

*^4^Department of Internal Medicine, Chung-Ang University Hospital, Seoul, Korea*

*^5^Interdisciplinary Program in Bioinformatics, Seoul National University, Seoul, Korea*

*^6^Department of Statistics, Seoul National University, Seoul, Korea*

*These authors equally contributed to this work.

**These co-corresponding authors equally contributed to this work.

**Supplementary Table 1. Characteristics of the patients according to the recurrence pattern**

| Variables | Local recurrence | Intrahepatic distant recurrence | *p* |
| --- | --- | --- | --- |
|  | (n = 5) | (n = 34) |  |
| Age, median (range) | 73 (52–77) | 59 (38–77) | 0.05 |
| Male, no. (%) | 5 (100.0) | 33 (97.1) | 1.00 |
| Etiology, no. (%) |  |  |  |
| Viral | 5 (100.0) | 30 (88.2) | 1.00 |
| Non-viral | 0 | 4 (11.8) |  |
| Initial treatment, no. (%) |  |  | 0.15 |
| Resection | 0 | 13 (38.2) |  |
| Locoregional therapies | 5 (100) | 21 (61.8) |  |
| RFA/TACE | 2/3 | 14/7 |  |
| FIB-4, no. (%) |  |  | 1.00 |
| ≤3.25 | 3 (60.0) | 21 (61.8) |  |
| >3.25 | 2 (40.0) | 13 (38.2) |  |
| Platelet, ×10^3^/mL, median (range) | 153 (44–249) | 145 (32–228) | 0.89 |
| ALT, IU/L, median (range) | 39 (16–165) | 37 (14–139) | 0.70 |
| Tumor number, median (range) | 1 (1–2) | 1 (1–2) | 0.50 |
| Maximal tumor size, median (range) |  |  | 0.73 |
| BCLC stage |  |  | 1.00 |
| 0 | 2 (40.0) | 11 (32.4) |  |
| A | 3 (60.0) | 23 (67.6) |  |
| Microvascular invasion* | - | 4 (30.8) |  |
| Encapsulation* | - | 9 (69.2) |  |
| Major Edmondson–Steiner grade* |  |  |  |
| I, II | - | 7 (53.8) |  |
| III, IV | - | 6 (46.2) |  |
| Worst Edmondson–Steiner grade* |  |  |  |
| I, II | - | 5 (38.5) |  |
| III, IV | - | 8 (61.5) |  |
| Serum AFP, ng/mL, median (range) |  |  |  |
| Pre-treatment | 5.8 (2.2–40.8) | 14.6 (1.8–4210) | 0.47 |
| Post-treatment | 5.3 (1.9–26.8) | 5.3 (1.9–127.2) | 0.95 |
| Difference | 1.8 (-0.5–14.0) | 2.9 (-24.5–4204.1) | 0.45 |
| Serum fibronectin, ng/mL, median (range) |  |  |  |
| Pre-treatment | 2.1 (1.6–22.6) | 4.1 (1.4–18.2) | 0.19 |
| Post-treatment | 2.8 (1.5–14.5) | 5.9 (1.5–16.4) | 0.07 |
| Difference | 0.06 (-1.1–8.2) | -1.0 (-10.6–3.2) | 0.10 |
| Median time to recurrence, months (range) | 10.3 (9.7–31.9) | 18.6 (6.6–40.5) | 0.32 |

* Assessed in patients who underwent resection.

Abbreviations: AFP = α-fetoprotein, ALT = alanine aminotransferase, BCLC = Barcelona Clinic Liver Cancer, HBV = hepatitis B virus, HCV = hepatitis C virus, TACE = transarterial chemoembolization

**Supplementary Table 2. Univariate logistic regression analyses for recurrence of hepatocellular carcinoma after excluding subjects with local tumor progression**

| Variables | Odds ratio | *p* |
| --- | --- | --- |
| Age | 0.96 | 0.12 |
| Gender |  |  |
| Female | - | - |
| Male | 18.86 | 0.006 |
| Treatment |  |  |
| Resection | - | - |
| Ablation therapy | 1.04 | 0.94 |
| TACE | 1.19 | 0.78 |
| Etiology |  |  |
| HBV | - | - |
| HCV | 2.77 | 0.26 |
| Alcohol | 0.69 | 0.68 |
| Others | 1.39 | 0.75 |
| FIB-4 |  |  |
| ≤3.25 | - | - |
| >3.25 | 0.81 | 0.66 |
| Platelet, ×10^3^/μL | 1 | 0.30 |
| ALT, IU/L | 1 | 0.52 |
| Tumor number | 0.80 | 0.67 |
| Maximal tumor size | 1.12 | 0.49 |
| BCLC stage |  |  |
| 0 | - | - |
| A | 1.08 | 0.87 |
| Microvascular invasion* | 0.64 | 0.56 |
| Encapsulation* | 0.77 | 0.78 |
| Major Edmondson–Steiner grade* |  |  |
| I, II | - | - |
| III, IV | 1.37 | 0.56 |
| Worst Edmondson–Steiner grade* |  |  |
| I, II | - | - |
| III, IV | 0.98 | 0.96 |
| AFP, ng/mL |  |  |
| Pre-treatment | 1 | 0.64 |
| Post-treatment | 1.04 | 0.23 |
| Difference | 1 | 0.64 |
| Fibronectin (log value) |  |  |
| Pre-treatment | 0.56 | 0.09 |
| Post-treatment | 1.15 | 0.67 |
| Difference^†^ | 0.006 | <0.001 |

* Assessed in patients who underwent resection.

^†^ defined by subtracting log-transformed post-treatment fibronectin value from log-transformed pre-treatment value.

Abbreviations: AFP = α-fetoprotein, ALT = alanine aminotransferase, BCLC = Barcelona Clinic Liver Cancer, TACE = transarterial chemoembolization

**Supplementary Table 3. Multivariate logistic regression analysis results after excluding subjects with local tumor progression**

| Variables | Odds ratio | *p* | AIC | AUC |
| --- | --- | --- | --- | --- |
| Male | 25.41 | 8.6$\times$10^-3^ | 71.47 | 0.87 |
| Log(fibronectin)_Diff_ | 3.7$\times$10^-3^ | 7.4$\times$10^-5^ |  |  |

Abbreviations: AIC = Akaike’s information criteria, AUC = area under the receiver-operating curve
